# Invariants and Other Structural Properties of Biochemical Models as a Constraint Satisfaction Problem

**DOI:** 10.1186/1748-7188-7-15

**Published:** 2012-05-29

**Authors:** Sylvain Soliman

**Affiliations:** 1 Equipe-Projet Contraintes, INRIA Paris-Rocquencourt, BP105, 78153 Le Chesnay Cedex, France

## Abstract

**Background:**

We present a way to compute the minimal semi-positive invariants of a Petri net representing a biological reaction system, as resolution of a Constraint Satisfaction Problem. The use of Petri nets to manipulate Systems Biology models and make available a variety of tools is quite old, and recently analyses based on invariant computation for biological models have become more and more frequent, for instance in the context of module decomposition.

**Results:**

In our case, this analysis brings both qualitative and quantitative information on the models, in the form of conservation laws, consistency checking, etc. thanks to finite domain constraint programming. It is noticeable that some of the most recent optimizations of standard invariant computation techniques in Petri nets correspond to well-known techniques in constraint solving, like symmetry-breaking. Moreover, we show that the simple and natural encoding proposed is not only efficient but also flexible enough to encompass sub/sur-invariants, siphons/traps, etc., i.e., other Petri net structural properties that lead to supplementary insight on the dynamics of the biochemical system under study.

**Conclusions:**

A simple implementation based on GNU-Prolog's finite domain solver, and including symmetry detection and breaking, was incorporated into the BIOCHAM modelling environment and in the independent tool Nicotine. Some illustrative examples and benchmarks are provided.

## 1 Background

### 1.1 Introduction

Reaction models like those of reactome.org, KEGG pathway database [1] or biomodels.net represent a growing part of Systems Biology especially for metabolic or signalling pathways, cell-cycle and more generally post-genomic regulation systems. They build on established standards like BioPAX or SBML [2] to facilitate the exchange and comparison of models and benefit from a large number of available tools, especially ODE integration based simulators.

The use of Petri nets to represent those models, taking into account the difference between compounds and reactions in the graph, and make available various kinds of analyses is quite old [3], however it remains somehow focused towards mostly qualitative and structural properties. Some have been used for module decomposition, like (I/O) T-invariants [4,5], related to dynamical notions of elementary flux modes [6]. However, there is, to our knowledge, very little use of P-invariant computation, which provides both qualitative information about some notion of module related to the "life cycle" of compounds, and quantitative information related to conservation laws - each P-invariant defines a conserved moiety of the obtained ODE system, whatever the rate laws - and Jacobian matrix singularity - induced by any P-invariant since it defines a linear dependency between variables. Conservation law extraction is actually already provided by a few tools, but then using numerical methods, based on the quantitative view of the model, and not integer arithmetic (as in direct P-invariant analysis).

We present here a very simple way to incorporate invariant computation in an existing biological modelling tool, using constraint programming with symmetry detection and breaking. We compare it to other approaches and evaluate it, for the case of P-invariants, on some examples of various sizes, like the MAPK cascade models of [7] and [8]. This experimentation is done through an implementation of the described method in the BIOCHAM modelling environment [9,10], and in the independent tool Nicotine. We benchmark the efficiency against state of the art Petri net tools on various models. Finally we show that the presented approach allows to compute, within the same framework, other interesting structural properties like sub/sur-invariants or siphons/traps, bringing even more insight into the dynamics of the biochemical system under study.

### 1.2 Petri net view of a reaction model

A Petri net is a bipartite oriented (weighted) graph of transitions, usually represented as square boxes, and places, usually represented as circles, that defines a (actually not unique) transition relation on *markings *of the net, i.e., multisets of tokens associated to places. The relation is defined by *firings *of transitions, i.e., when there are tokens (as many as the weight of the incoming arc) in all pre-places of a transition, they can be consumed and as many tokens as the weight on the outgoing arc are added to each post-place. The classical Petri net view of a reaction model is simply to associate biochemical *species to places *and biochemical *reactions to transitions*.

**Figure 1:**
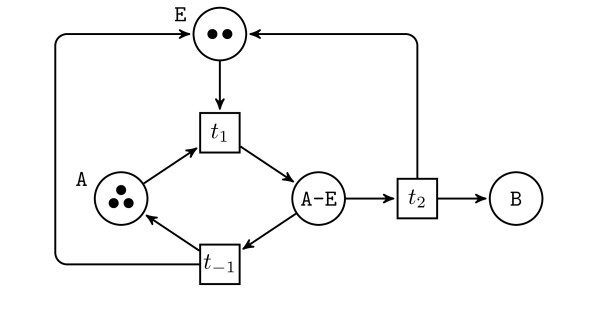
Petri Net

**Example 1 ***For instance the enzymatic reaction written (in BIOCHAM-like syntax)*, A + E ⇔ A-E ⇒ B + E* corresponds to the following Petri net *(Figure1)

*In this Petri net, starting from a marking with at least one token in A and in E, one can remove one of each to produce one token in A-E (firing of t*_1_*) and then either remove it to add again one token to A and one to E (firing of t*_-1_*), or to add one B and one E (firing of t*_2_*)*.

P (resp. T) invariants are defined, as usual, as vectors *V *representing a multiset of places (resp. of transitions) such that *V *· *I *= 0 (resp. *I *· *V *= 0) where *I *is the incidence matrix of the Petri net, i.e. *I_ij _*is the number of arcs from transition *i *to place *j*, minus the number of arcs from place *j *to transition *i*. Intuitively, a P-invariant is a multiset representing a weighting of the places and such that any such weighted marking remains invariant by any firing; a T-invariant represents a multiset of firings that will leave invariant any marking (see also section 2.1). As explained in the introduction, for reaction models these invariants are used for flux analysis, variable simplification through conservation law extraction, module decomposition, etc. Note that we are concerned with the classical invariant problem and thus restrict our study to integer weights. This is an important difference with respect to the aforementioned flux analyses but it arises from the fact that the biochemical models we studied did not come from metabolism but from the modelling of signal transduction pathways, cell cycle, circadian rhythm, etc. In all these cases the stoichiometry was integer and, for instance, the extracted conservation laws will include only integer number of molecules.

### 1.3 Related work

To compute the invariants of a Petri net, especially if this computation is combined with other Petri net analyses, like sinks and sources, traps, deadlocks, etc. the most natural solution is to use a Petri net dedicated tool like INA, PiNA, or Charlie for instance through the interface of Snoopy [11], which allows the import of SBML models as Petri nets. Standard integer methods like Fourier-Motzkin elimination will then provide an efficient means to compute P or T-invariants (see for instance [12] for a review). These methods however generate lots of candidates which are afterwards eliminated and also need to incorporate some means (like equality class definition) to avoid combinatorial explosion at least in some simple cases, as explained in Section 2.2.

Another way to extract the minimal semi-positive invariants of a model is to use one of the software tools that provide this computation for biological systems, generally as "conservation law" computation, and based on linear algebra methods like QR factorization [13]. This is the case for instance of the METATOOL [14] and COPASI [15] tools. The idea is to use a linear relaxation of the problem, which suits well very big graphs, but needs again *a posteriori *filtering of the candidate solutions. Moreover, these methods do not incorporate any means of symmetry elimination (see Section 2.2). A recent technique for elementary mode computation relies on Mixed Integer Programming (MIP) [16] and is thus quite similar in theory to the ideas of thus article, however it is tailor-made for elementary modes whereas for invariants pure Integer Programming would be enough, it is focused around the computation of a partial basis of these modes, which is an important problem but not the focus in this article, and - once again - it does not incorporate any symmetry breaking.

Finally, the most recent developments in invariant computation rely on a symbolic encoding through Binary decision Diagrams [17]. The tools based on this technique can prove quite efficient and are not unrelated to the symbolic encoding we present here through constraints. However they do not seem to integrate symmetry detection, also rely on filtering for minimality and thus, though they provide a symbolic solution very fast in some cases, might also benefit from some of the ideas we present. See section 2.5 for a more precise evaluation.

## 2 Results and Discussion

### 2.1 Finding invariants as a Constraint Satisfaction Problem

We will illustrate our new method for computing the invariants with the case of P-invariants (but T-invariants, being dual, would work in the same fashion). Consider a Petri net with *p *places and *t *transitions, these transitions represent reactions *L_i _*→ *R_i_*, where *L_i _*encodes the stoichiometry of the reactants as a vector over places, and *R_i _*the same for the products of the reaction. A P-invariant is a vector $V\in {\mathbb{N}}^{p}$ s.t. *V^T ^*· *I *= 0, i.e. ∀1 ≤ *i *≤ *t V *· *L_i _*= *V *· *R_i_*. Since those vectors all live in ${\mathbb{N}}^{p}$, it is quite natural to see this as a Constraint Satisfaction Problem (CSP) [18-20] with *t *(linear) equality constraints on *p *finite domain (FD) variables.

**Example 2 ***Using the Petri net of Example 1 we have:*

$$\begin{array}{llll} \text{A}+\text{E} & \Rightarrow \text{A}-\text{E}\; & & \;\\ \text{A}-\text{E} & \Rightarrow \text{A}+\text{E}\; & & \;\\ \text{A}-\text{E} & \Rightarrow \text{B}+\text{E}\; & & \;\\ & \; & \end{array}$$

This results in the following equations:

(1) A+E=AE

(2) AE=A+E

(3) AE=B+E

*where obviously equation (2) is redundant*.

The task is actually to find invariants with minimal support, with respect to set inclusion (a linear combination of invariants belonging to ${\mathbb{N}}^{p}$ also being an invariant), i.e., having as few non-zero components as possible, these components being as small as possible, but of course non trivial, we thus add the constraint that *V *· **1 **> 0.

**Example 3 ***In our running example we thus add A *+ *E *+ *AE *+ *B *> 0.

Now, to ensure minimality the labelling is invoked from small to big values. This means that for each variable, if an enumeration remains necessary after constraint propagation, values are tried in an increasing order starting at 0. This is closely related to the enumeration strategy used in the mixed integer programming method of [16] that allows them to look for *shortest *elementary modes. Such a restriction in the construction of the basis might thus also be possible in our approach. Then, a branch and bound procedure is wrapped around this search for solutions, maintaining a partial base  of P-invariant vectors and adding the constraint that a new vector *V *is solution if $\forall B\in B\prod_{B_{i}\neq 0}V_{i}=0$, which means that its support is not bigger than that of any vector of the base.

Unfortunately, even with the last constraint, no search heuristic was found that makes removing subsumed P-invariants unnecessary. Thus, if a new vector is added to , previously found vectors with a bigger support must be removed. Section 2.6 will demonstrate other structural properties for which this step is not necessary.

The algorithm can be summarized as follows:

**Algorithm 1 **Minimal invariants computation

1: post the CSP for invariant V: ∀1 ≤ *i *≤ *t V *· *L_i _*= *V *· *R_i _*and *V *· **1 **> 0

2: **repeat**

3:    find a solution, enumerating from low to high

4:    add the solution to the basis

5:    remove non-minimal invariants from the basis if there are any

6:    post the new constraint $\forall B\in B\prod_{B_{i}\neq 0}V_{i}=0$

7: **until **no solution found

8: expand symmetrical solutions of 

This algorithm was implemented directly into Nicotine^1 ^and then added to BIOCHAM [9], which are both programmed in GNU-Prolog, and allowed for immediate testing.

**Example 4 ***In our running example we find two minimal semi-positive P-invariants:*

• *E *= *AE *= 1 *and A *= *B *= 0

• *A *= *B *= *AE *= 1 *and E *= 0

### 2.2 Equality classes

The problem of finding minimal semi-positive invariants is clearly EXPSPACE-hard since there can be an exponential number of such invariants. For instance the model given in Example 5 (described in [12] among others, and called "classic X-Y" in [17], where × is the number of places between each pair of transitions and Y the number of transitions) has 2*^n ^*minimal semi-positive P-invariants (each one with either *A_i _*or *B_i _*equal to 1 and the other equal to 0).

**Figure 2:**
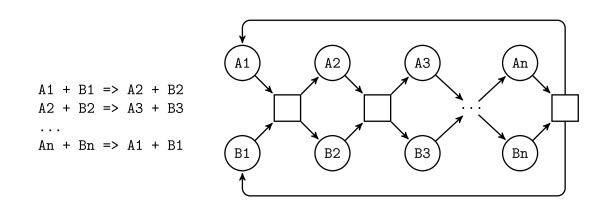
Example 5

**Example 5 (Classic 2-n) **(Figure2)

A first remark is that in this example, there is a variable symmetry between all the pairs (*A_i_*, *B_i_*) of variables corresponding to places. This symmetry is easy to detect (purely syntactical) and can be eliminated through the usual ordering of variables, by adding the constraints *A_i _*≤ *B_i_*.

This classical CSP optimization is enough to avoid most of the trivial exponential blow-ups and corresponds to the initial phase of *parallel places *detection and merging of the equality classes optimization [21] for the standard Fourier-Motzkin algorithm. Note however that in that method, classes of equivalent variables are detected and eliminated before and *during *the invariant computation, which would correspond to local symmetry detection and was not implemented in our prototype.

Moreover, in [21], *equality class *elimination is done through replacement of the symmetric places by a representative place. The full method reportedly improves by a factor two the computation speed. Even if in the context of the original article this is done only for ordinary Petri nets (Petri nets where the weights are only 0 or 1), we can see that it can be even more efficient to use this replacement technique in our case:

Example 6

...

A + B ⇒ 4*C

...

*Instead of simply adding A *≤ *B to our constraints, which will lead to 3 solutions when C *= 1 *before symmetry expansion: *(*A*, *B*) ∈ {(0, 4), (1, 3), (2, 2)}, *replacing A and B by D will reduce to a single solution D *= 4 *before expansion of the subproblem A *+ *B *= *D*.

This partial detection of independent subproblems, which can be seen as a complex form of symmetry identification, can once again be done syntactically at the initial phase, and can be stated as follows: replace ∑_*i *_k_i _* *A_i _*by a single variable *A *if all the *A_i _*occur only in the context of this sum i.e., in our Petri net all pre-transitions of *A_i _*are connected to *A_i _*with *k_i _*edges and to all other *A_j _*with *k_j _*edges and same for post-transitions. For a better constraint propagation, another intermediate variable can be introduced such that *A *= *gcd*(*k_i_*) · *A*'. In our experiments the simple case of *parallel places *(i.e., all *k_i _*equal to 1 in the sum) was however the one encountered most often.

### 2.3 Example, the MAPK Cascade

The MAPK signal transduction cascade is a well studied system that appears in lots of organisms and is very important for regulating cell division [22]. It is composed of layers, each one activating the next, and in detailed models shows two intertwined pathways conveying EGF and NGF signals to the nucleus.

A simple MAPK cascade model, that of [23] without scaffold, is used here as an example to show the results of P-invariant computation.

**Figure 3:**
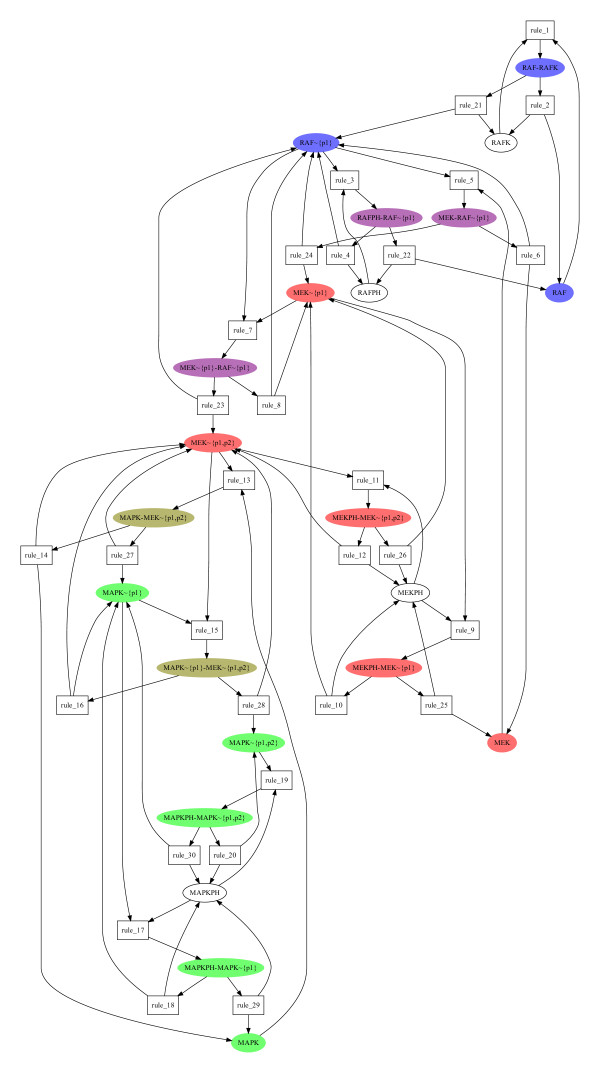
**Some conservation laws of the MAPK model of **[23]. 3 of the 7 P-invariants found in the MAPK cascade model of [23]. The blue one (RAF), the pink one (MEK) and the green one (MAPK) with intersections in purple (blue+pink) and khaki (pink+green).

Seven minimal semi-positive P-invariants are found almost instantly. Intuitively, they represent the different levels of the cascade (i.e., RAFK, RAF, MEK and MAPK) and the corresponding phosphatases (RAFPH, MEKPH and MAPKPH). The use of those P-invariants as visual *modules*, as depicted in Figure 3 is quite similar to one part of the approach of [24] to make biochemical systems more easy to grasp. The full list is given in Table 1.

**Table 1:** P-invariants of the MAPK cascade model of [23]

RAFK, RAF-RAFK
RAFPH, RAFPH-RAF~{p1}

RAF, MEK-RAF~{p1}, RAF-RAFK, RAFPH-RAF~{p1}, MEK~{p1}-RAF~{p1}, RAF~{p1}

MEKPH, MEKPH-MEK~{p1}, MEKPH-MEK~{p1, p2}

MEK, MAPK-MEK~{p1, p2}, MEK-RAF~{p1}, MEKPH-MEK~{p1}, MEKPH-MEK~{p1, p2}, MAPK~{p1}-MEK~{p1, p2}, MEK~{p1}-RAF~{p1}, MEK~{p1}, MEK~{p1, p2}

MAPKPH, MAPKPH-MAPK~{p1}, MAPKPH-MAPK~{p1, p2}

MAPK, MAPK-MEK~{p1, p2}, MAPKPH-MAPK~{p1}, MAPK~{p1, p2} MAPK~{p1}-MEK~{p1, p2}, MAPK~{p1}, MAPKPH-MAPK ~{p1, p2},

Full list of the P-invariants of the MAPK cascade model of [23]

In the next section other examples are used as benchmarks of this method, they are all much bigger than this one, which had only about 30 compounds, however note that one of those is still a model of the MAPK signalling cascade.

Note that these 7 P-invariants define 7 algebraic conservation rules (i.e., mass conservation) and thus decrease the size of the corresponding ODE model from 22 variables and equations to only 15.

### 2.4 Evaluation on other biochemical examples

Schoeberl's model is a more detailed version of the MAPK cascade, which is quite comprehensive [8], but too big to be studied by hand. It can however be easily broken down into fourteen more easily understandable units formed by P-invariants, as shown in Table 2, along other examples representing amongst the biggest reaction networks publicly available.

**Table 2:** Minimal semi-positive P-invariant computation on bigger models of biochemical reaction networks

Model	transit.	places	P-invar.	time (s)	Invariant size
Schoeberl's MAPK [8]	125	105	13	0.53	from 2 to 44

Calzone et al. E2F/Rb [31]	~500	~400	79	18	from size 1 (EP300) to about 230 (E2F1 box)

Kohn's map [32]	~800	~500	70	171	from size 1 (Myt1) to about 200 (pRb or cdk2)

Minimal semi-positive P-invariant computation on bigger models of biochemical reaction networks

All the curated models in the September 2010 release of biomodels.net were also tested and none of them required more than 1s to compute all its minimal P-invariants.

We could not compare our results with those provided in [13] since the models they use, coming from metabolic pathways flux analyses, do not have an integer stoichiometry matrix, however the examples of Table 2 show the feasibility of P-invariant computation by constraint programming for quite big networks. Note that for networks of this size, the upper bound of the domain of variables had to be set manually. It was actually set to the value 8, which is about the double of the maximum value in all the biological models we have encountered up to now. The only over-approximation of the upper bound found was the product of the *l.c.m. *of stoichiometric coefficients of each reaction, which explodes really fast and leads to unnecessarily long computation. The manual bound results in a loss of completeness, but it is not enforced either by QR-factorization methods, and does not seem to miss anything on real life examples.

Though they are not specifically suited for this task (i.e., finding integer invariants), we tried some of the most well known Elementary Flux Modes computing packages on these examples. METATOOL [14] and efmtool [25] were chosen, since both can be run as Matlab packages. The results are not included in Table 2 but are summarized with the non-biochemical examples of next section.

### 2.5 Non-biochemical benchmarks

Even if our main purpose is to use the insight on the dynamics gained from the structural properties computed by our CSP, an evaluation of the proposed method on non-biochemical models remains of interest.

The literature on invariant computation is quite large, however there does not seem to exist any standardized benchmark. Each author selects some examples with different properties (see for instance [12] from which only a few examples are used in [17], even though it is cited as reference) and few reuse the previously published sets of examples.

Moreover, even when the software used in these articles is available, usually only binary implementations are available, and only for some specific architectures and through a specific request process. In some cases none is provided at all.

Therefore, using a machine comparable in specifications, we chose to reuse the data published in the most recent work, that of Ciardo et al. [17]. Since we had to re-encode ourselves the selected examples, only a subset of their benchmarks is covered, namely the classical dining philosophers problem [26], the standard exponential invariant case [12] and the circular trains [27]. These seem to cover the whole range of different schemes appearing in [17].

Note that there are usually many symmetries in these parametric examples and thus that a more powerful (or manual) symmetry detection would be called for in these specific cases. Nevertheless, since (intracellular) biochemical systems usually do not generate such structure, we did not push further the integration of more advanced symmetry detection/breaking in our tools.

All the models used for the biochemical and non-biochemical benchmarks can be found at: http://contraintes.inria.fr/~soliman/nicotine_data/

METATOOL's "CONSERVATION RELATIONS" were used when possible, but that only allows to find - as expected - 91 out of the 10 billion invariants for the classic example, in 0.33s. Models were thus *transposed *such that METATOOL and efmtool's EFM search correspond to P-invariant computation. Transposed models appear with a 'b' ending in the data repository. efmtool was given the SBML files as input whereas some .dat files were generated for METATOOL. For all the examples of this section as well as Kohn's map, METATOOL gave the error message "Cannot sort modes with more than 52 rows" that was interpreted as some kind of "out of memory" error. For efmtool, in the same cases (all examples of this section plus Kohn's map) the computation was stopped after 10 minutes or more, with messages like "iteration 43/116: 224850 modes, dt = 2040206ms." that were interpreted as overtime. Note however that as already stated, these packages do not focus on *integer *stoichiometric matrices and thus have a much broader scope that might explain their poor performance on our benchmarks.

The results are presented in Table 3, where as in [17] "om" represents an out-of-memory error, and "ot" an overtime. "na" was used when conservation relations are strictly fewer than P-invariants. The results seem to indicate that a constraint-based approach fares reasonably well, usually in the same order of magnitude as some purely symbolic encoding via decision diagrams [17], whereas the solutions of the CSP are explicit. Even in the case where finding explicit solutions revealed too costly (classic 10-10, which has 10^10 ^minimal P-invariants), one can stop the computation before symmetry expansion and get an answer in a reasonable time.

**Table 3:** Minimal semi-positive P-invariant computation on general (non-biochemical) benchmarks of the literature

model	BDD V2	BDD V4	GreatSPN	Nicotine	Metatool CR	Metatool EFM	efmtool
trains 10-10	4.81	om	0.03	3.26	na (20)	om	ot
classic 10-10	0.01	0.01	ot	0.15	na (91)	om	ot
philo 30	1.04	0.01	0.01	2.68	3.04	om	ot

Minimal semi-positive P-invariant computation on general (non-biochemical) benchmarks of the literature. Times are given in seconds. BDD V2 and V4 (implicit) and GreatSPN (explicit) performances as per [17]. Note that for the *classic *example, time was measured for Nicotine before symmetry expansion (semi-implicit) since there are 10^10 ^explicit solutions.

The CSP approach can therefore be seen as a kind of intermediate between purely implicit (i.e., solutions encoded, for instance as a decision diagram, and needing to be decoded to be displayed) and purely explicit methods. It also remains very flexible as next section will prove and could incorporate many more optimizations (variable ordering heuristics, more symmetry elimination, etc.) at a quite low cost.

All the 80 Petri nets of http://www.petriweb.org/ were also tested. Only one took more than 1s: model 1516, which took about 3s to compute 1133 minimal P-invariants. Since we do not have data for the other approaches on these models they were not added to the table of results but they confirm the feasibility and generality of our approach.

We think that the structure of this kind of net is however very different (average degree, arc weights, etc.) from that of usual biochemical reaction models and intend to explore this distinction further in the future.

### 2.6 Generalizing the approach to other structural properties

An interesting feature of the presented method is that it is actually flexible enough to encompass other structural properties than place or transition invariants. This is, to our knowledge, not the case of other alternative methods.

If for the Petri net of Example 1 one obtained the constraints shown in Example 2 to compute P-invariants, one can notice that they can easily be adapted to compute sur- or sub-invariants, i.e., weighted sums that can only grow (resp. decrease) during the evolution of the system (see [28], for instance, for a formal definition). Indeed the following CSP describes exactly all the sub-invariants of the system and is obtained in the same manner but with ≤ instead of =.

**Example 7 ***Using the Petri net of Example 1:*

$$\begin{array}{llll} \text{A}+\text{E} & \Rightarrow \text{A}-\text{E}\; & & \;\\ \text{A}-\text{E} & \Rightarrow \text{A}+\text{E}\; & & \;\\ \text{A}-\text{E} & \Rightarrow \text{B}+\text{E}\; & & \;\\ & \; & \end{array}$$

results in the following FD constraints:

(4) A+E≤AE

(5) AE≤A+E

(6) AE≤B+E

Sur-invariants would be obtained with ≥ instead of ≤.

Now, getting a basis of minimal sub/sur-invariants can be done with the same branch and bound technique used for invariants, allowing to obtain information on pools of species of the biochemical system that, for instance, never increase during any ODE simulation.

One can go slightly farther and once again reuse the same machinery, including symmetry breaking, to compute siphons and traps of the Petri net (see [29] for definition and example of use in biology). This time a boolean CSP is obtained with the following constraints for the example of traps:

**Example 8 ***Using the Petri net of Example 1 we obtain the following boolean constraints:*

(7) A∨E⇒AE

(8) AE⇒A∨E

(9) AE≤B∨E

To compute siphons one simply need to reverse ⇒ into ⇐.

Note that in the boolean domain, the support minimality can be imposed by enumerating in increasing (lexicographic) order, there is no need for any a *posteriori *check of minimality (step 5 of Algorithm 1). The algorithm thus becomes:

**Algorithm 2 **Minimal traps computation

1: post the CSP for trap V

2: **repeat**

3:    find a solution, enumerating from low to high

4:    add the solution to the basis

5:    post the new constraint $\forall B\in B\prod_{B_{i}\neq 0}V_{i}=0$

6: **until **no solution found

7: expand symmetrical solutions of 

This computation of traps and siphons can actually bring information about the dynamics of the model, including temporal logic formulae that it satisfies^2^, together with other structural properties [4,30] they provide an interesting toolkit to analyze structurally the dynamics of a Systems Biology model.

## 3 Conclusion

P-invariants of a biological reaction model are not so difficult to compute in most cases. They carry information about conservation laws that are useful for efficient and precise dynamical simulation of the system, and provide some notion of module, which is related to the life cycle of molecules. T-invariants are already used more commonly, and get more and more focus recently.

We introduced a new method to efficiently compute P and T-invariants of a reaction network, based on FD constraint programming. It includes symmetry detection and breaking and scales up well to the biggest reaction networks found. Completeness is lost on the biggest examples but we still look for a better upper bound on domains to restore it.

The idea of applying constraint based methods to classical problems of the Petri net community is not new, but seems currently mostly applied to the model-checking. We argue that structural problems (invariants, sinks, attractors, etc.) can also benefit from the know-how developed for finite domain CP solving, like symmetry breaking, search heuristics, flexibility, etc. and thus intend to generalize our approach to other problems of this category.

## Competing interests

The author declares that they have no competing interests.

## Endnotes

^1^http://contraintes.inria.fr/~soliman/nicotine.html

^2^This is the topic of a paper currently submitted to the CMSB 2011 conference. Depending on the outcome, a reference or a short explanation will be added.
